# The Significance of Neuropilins in Gastrointestinal Cancers

**DOI:** 10.3390/ijms26104937

**Published:** 2025-05-21

**Authors:** Kinga Królikowska, Natalia Kurman, Katarzyna Błaszczak, Sławomir Ławicki, Monika Gudowska-Sawczuk, Monika Zajkowska

**Affiliations:** 1 Department of Population Medicine and Lifestyle Diseases Prevention, The Faculty of Medicine, Medical University of Bialystok, 15-269 Bialystok, Polandslawicki@umb.edu.pl (S.Ł.); 2 Department of Biochemical Diagnostics, Medical University of Bialystok, Waszyngtona 15A St., 15-269 Bialystok, Poland; monika.gudowska-sawczuk@umb.edu.pl; 3 Department of Neurodegeneration Diagnostics, Medical University of Bialystok, Waszyngtona 15A St., 15-269 Bialystok, Poland

**Keywords:** GI cancers, colorectal cancer, gastric cancer, pancreatic cancer, liver cancer, NRP

## Abstract

Cancers represent a significant global health concern, being among the most prevalent malignancies and contributing substantially to morbidity and mortality rates. Notably, colorectal, gastric, pancreatic, and liver cancers are the most frequently diagnosed among these malignancies. The pathogenesis of gastrointestinal (GI) cancers is multifactorial, encompassing a complex interplay of genetic predispositions, environmental exposures, and lifestyle choices. Despite advances in diagnostic approaches and therapeutic strategies, existing treatment modalities, particularly in the advanced stages of these cancers, remain ineffective. Recent research efforts have increasingly focused on the identification and characterization of novel biomarkers that could enhance both the detection and treatment of gastrointestinal cancers. One particularly promising area of investigation involves neuropilins (NRPs). NRPs are involved in essential biological processes such as angiogenesis, cellular migration, and tumor cell-microenvironment interactions, all of which promote tumor progression and contribute to the development of treatment resistance. Overexpression of neuropilins has been linked to poor prognosis in patients, implying that they could be useful in diagnosis and serve as targets for molecular treatment. Recent research also suggests that inhibiting neuropilin activity may slow tumor growth and inhibit angiogenic processes, opening up new possibilities for targeted therapeutic techniques in the treatment of gastrointestinal malignancies.

## 1. Introduction

Gastrointestinal cancers constitute a major category of malignancies globally, contributing significantly to morbidity and mortality rates. The most prevalent types within this group include colorectal, gastric, pancreatic, and liver cancers. According to epidemiological data, colorectal cancer (CRC) is the 3rd most frequent gastrointestinal malignancy and the 2nd most dominant origin of cancer-related death in this sector. Furthermore, it has also been noted that stomach tumor is the most prevalent disease in Asian countries, where it ranks among the most frequent origin of cancer-related deaths [1]. Colorectal, liver, pancreatic, and gastric cancers are gastrointestinal malignancies with different causes but similar characteristics. These cancers are commonly associated with a poor diet, smoking, obesity, and chronic inflammatory illnesses. Early symptoms are frequently ambiguous, and many diseases are typically detected at an advanced stage. Genetic mutations may also be a risk factor for colorectal and stomach cancer, but liver and pancreatic cancers are frequently linked to persistent viral infections (HBV, HCV) and metabolic disorders. Treatment is determined on the stage of the disease; for advanced gastrointestinal cancer, surgery, chemotherapy, or targeted medicines are frequently employed. Early detection is critical for improving the prognosis [2]. Despite substantial advancements in diagnostic and therapeutic approaches, the efficacy of currently available methods remains constrained, particularly in the advanced stages of the disease. Conventional tumor biomarkers, including carcinoembryonic antigen (CEA) and the carbohydrate antigen (CA19-9), exhibit limitations in specificity and sensitivity. These deficiencies impede their effective utilization in monitoring disease progression and predicting clinical outcomes [3]. As a result, finding novel biomarkers that can substantially develop the effectiveness of malignancy detection and treatment has emerged as a crucial emphasis in medical research. Neuropilins (NRPs) are transmembrane proteins that act as coreceptors for a number of growth factors, including semaphorins and vascular endothelial growth factor (VEGF), are one particularly interesting field of research. This field of research has the possibility to enhance our knowledge of cancer biology and therapeutic approaches [4]. Neuropilins are integral components in various physiological processes, including angiogenesis, neurogenesis, and cell migration. In normal physiological conditions, the expression of neuropilins is subject to precise regulatory mechanisms. However, under pathological conditions, particularly cancer, significant changes in their expression levels and functional roles might be detected. These modifications may contribute to tumor growth and metastasis, highlighting the importance of neuropilins in both healthy and diseased tissues [5]. Neuropilins have an important function in altering the interaction between tumor cells and their microenvironment, which may increase tumor cell survival and contribute to resistance to traditional therapeutic interventions. Furthermore, neuropilins are involved in the regulation of growth factor signaling pathways and influence the interactions between tumor cells and immune cells. These mechanisms may facilitate the evasion of tumor cells from immune surveillance and therapeutic control, thereby promoting tumor progression and metastasis [6]. In cancer biology, neuropilins promote tumor growth in a variety of ways. Their capacity to activate angiogenesis increases the flow of oxygen and nutrients to the growing tumor, which significantly accelerates tumor growth [7]. Furthermore, neuropilins regulate tumor cell motility and invasion, which increases their metastatic potential. The interactions of neutropilins with the tumor microenvironment, as well as their impact on immunosuppression, underline their potential as therapeutic targets and prognostic biomarkers in gastrointestinal tumors [8]. Moreover, research conducted so far shows that neuropilin overexpression in malignancies is associated with a poorer prognosis for patients, emphasizing their importance in laboratory diagnostics and clinical oncology. Further investigations have shown that inhibiting neuropilins with molecular inhibitors can reduce tumor cell proliferation and limit tumor angiogenesis. These findings suggest the potential utility of these proteins in laboratory diagnostics and neuropilin-targeted therapies in the management of cancer [9].

## 2. Neuropilins

**Figure 1:**
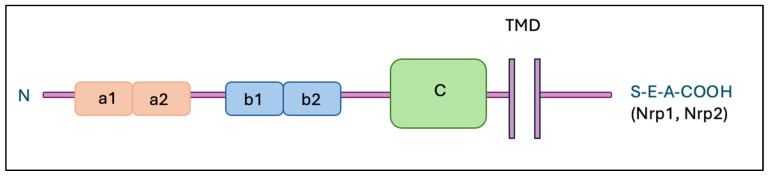
Structure of neuropilins. Abbreviations: Nrp-1—neuropilin 1; Nrp-2—neuropilin 2; TMD—transmembrane domain; a1, a2, b1, b2—extracellular domains.

Neuropilins, such as Nrp-1 and Nrp-2, are transmembrane glycoproteins that play critical roles in ontogenesis, immunology and oncology. Both neuropilins have a similar structure (shown in Figure 1), consisting of an extracellular part containing domains such as a1/a2, b1/b2, and c, as well as a transmembrane part and a short cytoplasmic fragment [10].

Their isoforms are also known, including soluble forms (sNrp-1 and sNrp-2) and an alternate membrane variant of Nrp-2 (Nrp-2b). Expression of Nrp-1 and/or Nrp-2 has been found in different cell types, such as endothelial cells, neurons, pancreatic islet cells, hepatocytes, and tumor cells [11]. Nrp-1 predominates in arterial endothelial cells, while Nrp-2 is mainly present in endothelial cells of veins and lymphatic vessels. In the immunological system, Nrp-1 is found primarily on thymocytes [12], plasmacytoid dendritic cells [13], and regulatory T cells [14]. Neuropilins have the ability to bind VEGF family proteins and SEMA3; Nrp-1 binds primarily SEMA3A, while Nrp-2 selects SEMA3F. Both neuropilins also bind to different ligands within the VEGF family proteins. In addition, they show interactions with other ligands such as TGF-β1, HGF, PDGF, and FGF, which affects signal transduction and modulation of a variety of cellular processes [15,16]. Table 1 summarizes specific ligands for NRP receptors.

Neuropilins play a complex role in cancer development. They are often observed to be overexpressed in various tumor types, which may be connected with bad prognoses. They influence such processes as proliferation, migration, invasiveness, and the capability of malignant cells to metastasize. They have also been shown to be significant in tumor-associated angiogenesis and lymphangiogenesis [17]. Furthermore, neuropilins may contribute to anticancer treatment resistance and affect the immunological response in cancer. Neuropilins, with their ability to rapidly transport Nrp-1-bound compounds into cells, may become a therapeutic target for cancer treatment, allowing for more efficient drug transport into cancer cells [18]. This review specifically focuses on the emerging role of neuropilins as integrative biomarkers and therapeutic targets in gastrointestinal cancers, an area that has been underexplored. Neuropilins promote processes such as angiogenesis, metastasis, and invasion, which are very important processes during GI cancer development. While their overexpression correlates with poor prognosis and has made them promising therapeutic targets through approaches like NRP inhibitors, monoclonal antibodies, and CAR-T cell therapy, their broad biological functions also pose significant challenges for developing precise and effective treatments.

## 3. Neuropilins in Colorectal Cancer

Neuropilins (NRPs) are transmembrane receptors that serve crucial roles in many biological processes, including angiogenesis, cell migration, and tumor progression. Notably, neuropilin-1 (NRP-1) and neuropilin-2 (NRP-2) have been identified as significant regulators of the tumor microenvironment in colorectal cancer (CRC). The expression and functional implications of these receptors have been extensively investigated to assess their potential as diagnostic and prognostic biomarkers. Research has consistently demonstrated that both neuropilins are substantially overexpressed in cancer tissues when in comparison to normal colorectal mucosa, indicating their potential involvement in the pathophysiology of CRC [19]. High levels of NRP-1 have been linked with a more aggressive tumor cell phenotype, suggesting its potential importance as an indicator of disease progression [20]. In contrast, NRP-2, while less comprehensively characterized than NRP-1, is implicated in the pathogenesis of CRC. It contributes significantly to the control of epithelial-mesenchymal transition (EMT), thereby contributing to the spreading potential of malignancies [21]. Neuropilin expression is regulated at both transcriptional and epigenetic levels. Hypoxia and various growth factors, particularly vascular endothelial growth factor (VEGF), have a considerable influence on neuropilin expression [19,21]. One of the principal mechanisms by which NRP-1 and -2 contribute to tumor biology is through the induction of angiogenesis. NRP-1 functions as a coreceptor for VEGF, facilitating the amplification of signaling through vascular endothelial growth factor receptor 2 (VEGFR-2). This interaction promotes the enhanced formation of new blood vessels within the tumor microenvironment, thereby encouraging tumor development and progression [19]. NRP-2 has a similar function, but its role in angiogenesis is further linked to its effect on lymphangiogenesis, which may facilitate lymph node metastasis [22]. In addition, both neuropilins are engaged in the regulation of signaling pathways connected with adhesion, migration, and EMT, which increases the capacity of tumor cells for metastasis [19,22]. Numerous analyses have shown that enhanced expression of NRP-1 and NRP-2 in colorectal cancer tissues correlates with adverse patient prognosis, including a higher risk of metastasis and shorter overall survival [20,22]. Moreover, elevated levels of both neuropilins have been connected with greater tumor invasiveness and increased angiogenesis, leading them to be potential prognostic biomarkers and therapeutic targets [19]. Given the critical function of NRP-1 and NRP-2 in CRC growth, research is being conducted to determine the feasibility of inhibiting them pharmacologically. Preliminary studies reveal that NRP-1 and NRP-2 inhibitors can efficiently limit angiogenesis and reduce the tumor’s potential to metastasize, making them promising therapeutic targets [19,21]. These strategies, especially when combined with anti-VEGF therapy, have the potential to improve therapeutic efficacy and prognosis in colorectal cancer patients.

## 4. Neuropilins in Gastric Cancer

Over the past decade, numerous studies have explored the expression of the neuropilin-1 (NRP-1) protein in gastric cancer, examining its correlation with various clinicopathological features. This publication aims to systematically assess the relationship between NRP-1 protein expression and the clinicopathological characteristics associated with gastric cancer by reviewing the existing literature on this topic. The findings indicate that the positive expression rate of NRP-1 protein is significantly elevated in patients with tumors larger than 5 cm compared to those with tumors smaller than 5 cm. Additionally, higher expression levels are observed in patients classified as stages III–IV relative to those in stages I–II, as well as in cases of poor differentiation compared to those with good or moderate differentiation. Furthermore, NRP-1 protein expression is more prevalent in patients with lymph node metastases than in those without. However, no statistically significant associations were identified between NRP-1 protein expression and variables such as gender, age, clinical stage, or the Laurén classification system [23]. In contrast, NRP-1 has been identified as a critical mediator in axon guidance and angiogenesis, primarily through its interactions with semaphorins and the VEGF family of growth factors. Furthermore, evidence suggests that NRP-1 promotes tumor growth by engaging with various extracellular growth factors and their corresponding receptors, including hepatocyte growth factor (HGF) and its receptor c-Met, fibroblast growth factors (FGFs), VEGF, and transforming growth factor beta (TGF-β). The role of NRP-1 in tumor metastasis appears to be multifaceted. It facilitates endothelial cell migration induced by VEGF, enhances tumor cell migration and proliferation through autocrine signaling involving Semaphorin 3A (Sema 3A) and HGF/scatter factor signaling pathways. According to recent reports, Sema3A may play an antitumor role in gastric cancer by acting as an inhibitor of NRP-1 activity. The authors observed that overexpression of Sema3A significantly attenuated the proliferation, migration, and invasiveness of gastric cancer cells, and this effect was associated with the inhibition of the PI3K/Akt pathway and reduced NRP-1 expression [24]. Furthermore, NRP-1 was shown to directly interact with fibronectin-1 (FN1), which promotes epithelial-mesenchymal transition (EMT) in gastric cancer cells. Blocking NRP-1 resulted in the downregulation of EMT markers (e.g., N-cadherin, vimentin) and the upregulation of E-cadherin, suggesting that NRP-1 plays an important role in cancer progression and increased invasiveness [25]. Importantly, high levels of NRP-1 expression are associated with poor prognosis in patients with gastric adenocarcinoma, particularly in the intestinal subtype according to the Laurén classification [26].

Additionally, NRP-1 contributes to tumor growth by modulating the tumor microenvironment, which results in the increased organization of fibronectin fibrils. It also plays a role in maintaining the dedifferentiated phenotypes of tumor cells, preserving stem cell-like properties, and inducing immunosuppressive signaling within the tumor milieu. Specifically, NRP-1 has been shown to significantly influence the progression of gastric cancer by regulating tumor cell migration and invasion [27]. Research into the therapeutic application of monoclonal antibodies targeting NRP-1 has yielded promising results in the context of gastric cancer treatment. Ding et al. used a new anti-NRP-1 monoclonal antibody (anti-NRP-1 mAb) to investigate its potential anti-tumor effects on human gastric cancer cell lines in vitro and in vivo. These findings demonstrated that treatment with anti-NRP-1 mAb significantly inhibited the migration and invasion of BGC-823 cell lines, while maintaining cellular viability at concentrations below 150 µg/mL. Mechanistically, the action of the antibody was associated with the dephosphorylation of the Akt protein, suggesting an inhibition of the PI3K/Akt signaling pathway, which is vital for the migratory and invasive behaviors of malignant cells. Furthermore, in a mouse model of human gastric cancer xenografts, administration of anti-NRP-1 mAb resulted in a marked reduction in both tumor volume and weight, without exhibiting any discernible toxic effects. These results imply that the blockade of NRP-1 using specific monoclonal antibodies may represent a new and effective therapeutic strategy for gastric cancer, primarily through the inhibition of tumor cell migration and invasion via modulation of the PI3K/Akt signaling pathway [28]. Neuropilin 2 (NRP-2) has emerged as an important factor in the progression of gastric cancer. Empirical evidence indicates that elevated expression levels of NRP-2 correlate with increased anti-apoptotic mechanisms and enhanced metastatic potential of tumor cells. NRP-2 functions as a co-receptor for a variety of growth factors, including VEGF, TGF-β, and HGF, thereby modulating intercellular communication and the migratory behavior of cancer cells. These interactions facilitate the activation of critical signaling pathways, such as the PI3K/Akt, ERK, and MAPK pathways. The activation of these pathways subsequently promotes cellular proliferation, survival, and invasive capabilities of cancer cells, underscoring the pivotal role of NRP-2 in gastric cancer pathology [29]. Increased expression of neuropilin-2 (NRP-2) has been correlated with enhanced lymphangiogenesis, the process connected to the creation of new lymphatic vessels, which facilitates the dissemination of cancer cells to lymph nodes and distant organs. Inhibition of NRP-2 function through the application of specific monoclonal antibodies has been shown to result in a significant reduction in tumor-associated lymphangiogenesis, as well as a decrease in metastasis to lymph nodes and other distant sites [30]. Elevated levels of NRP-2 have been consistently correlated with disease progression and poorer prognostic outcomes in patients diagnosed with various malignancies, including prostate cancer, osteosarcoma, salivary gland carcinoma, breast cancer, neuroblastoma, and non-small cell lung carcinoma [29]. Currently, research is being conducted to clarify the mechanisms of action of NRP-2 and to develop targeted therapeutic strategies with the potential to treat variety of malignancies, including gastric cancer.

## 5. Neuropilins in Pancreatic Cancer

Unfortunately, it is obvious that pancreatic cancer is the deadliest cancer in the world. The main types of pancreatic cancers are pancreatic ductal adenocarcinoma (PDAC) and pancreatic endocrine tumors [31,32,33,34,35]. However, advanced-stage PDAC accounts for the majority of diagnosed pancreatic cancer cases. Due to the late detection stage and lack of effective treatment, the survival rate is less than 5% [35]. Accordingly, there is a need for innovative drugs and treatment options of pancreatic cancer [31,32]. Cancerous tumors are most often located in the head of the pancreas, and their diameter ranges from 2.5 to even 3.5 cm. PDAC is an infiltrating epithelial tumor with glandular differentiation in the pancreas [35]. Morphologically, it is characterized by severe fibrosis, referred to as tumor desmoplasia [36,37]. The most common cells around the tumor stroma are cancer-associated fibroblasts (CAFs). CAFs, together with different endothelial cells, constitute most of the tumor. Cancer cells have an eosinophilic, abundant cytoplasm and enlarged nuclei, with perineural invasion and infected blood and lymphatic vessels typically observed. In several cases, cystic changes may occur, which are caused by necrosis or dilatation of the pancreatic ducts [32]. It has been revealed that endothelial cells could change their character through endothelial-mesenchymal transition, which is a key mechanism leading to the development of fibroblasts in cancer [35,37]. Neuropilin-1 (NRP-1) was originally associated with the evolution of the nervous system, but increasing evidence indicates its important role in cancer, particularly in angiogenesis, chemoresistance and other aspects of malignant progression [35]. In healthy pancreatic tissue, neuropilins are found in small amounts, mainly in vascular endothelial cells. On the other hand, analyses of neuropilin expression studies have shown that their levels are significantly increased in PDAC tissue and tumor microenvironment, compared to healthy pancreatic tissue. This is related with cancer development and a negative outcome for the patient [33]. NRP-1 functions as a coreceptor for many growth factors, including vascular endothelial growth factor A (VEGF-A), fibroblast growth factor (FGF), hepatocyte growth factor (HGF), and transforming growth factor beta 1 (TGFβ1). In addition, NRP-1 has an ability to increase VEGF receptor signaling and pro-angiogenic activity, which indicates increased intratumoral angiogenesis and disease progression, and it is found on both endothelial and tumor cells [37]. Interestingly, it also actively binds TGFβ1, a key factor inducing endothelial to mesenchymal transition (EndMT), which has opened up a completely new area of research on oncogenic processes such as epithelial-to-mesenchymal transition (EMT) [33]. Given the significant role of NRP-1 in the progression of pancreatic cancer, scientists began to investigate its potential role in the treatment of this disease. It has been revealed that NRP-1 silencing reduces the canonical TGFβ signaling pathway and reduces the expression of profibrotic genes. In contrast, NRP-1 overexpression exacerbates TGFβ1-induced EndMT and increased TGFβ signaling and profibrotic gene expression. In vivo studies have proven that the loss of NRP-1 leads to decreased perfusion and tumor size, which is correlated with a decrease in the intensity of the EndMT process and fibrosis. The data obtained so far clearly indicate that increased NRP-1 signaling in tumors is correlated with increased angiogenesis in vivo. Nevertheless, both tumor and endothelial cells produce NRP-1 and other VEGF signaling molecules; therefore, the role of endothelial cells in tumor expression and activation cannot be ruled out [33,37]. As already mentioned above, PDAC is characterized by high desmoplasia and dense tumor stroma [37]. The information obtained suggests a fundamental for the development of combination therapy binding an NRP-1 antagonist with drugs used in standard treatment [18]. It was concluded that the tumor-penetrating peptide utilizes the NRP-1-regulated transcytosis transport pathway. This mechanism increases the efficiency of transcytosis of chemotherapeutics based on silica nanoparticles in the case of PDAC in cells expressing NRP-1. Hence, the NRP-1 peptide antagonist may offer greater potential for tumor penetration than the current treatment involving anti-NRP-1 antibodies [38]. Furthermore, immunotherapy using chimeric antigen receptor (CAR)-positive T cells enables them to identify and bind to cells that express a specific for NRP-1 antigen. As a result, targeting CAR-T cells to NRP-1 may represent promising therapeutic strategy for treating PDAC [39]. Additionally, it was found that NRP-1 and NRP-2 have very short intracytoplasmic domains that are dependent on scaffolding proteins to transmit biological signals. One such protein is the C-terminal protein GAIP (GIPC1) that is overexpressed in pancreatic cancer as well as being associated with poor survival. Studies conducted in mice have shown that knocking down NRP-1, NRP-2, or GIPC-1 alone has significant effects. On the other hand, only small additive effects could be detected after mixed knockdown and no counterregulation of the corresponding different genes. Therefore, it seems that inhibiting NRP-1 using polymeric nanoparticles to deliver specific siRNAs inhibits tumor growth or reduces proliferation, making it a promising approach for treating of PDAC [35]. In conclusion, despite NRP-1′s complicated role in endothelium and cancer cells, as well as its participation in tumor growth, it remains a prospective therapeutic target for PDAC.

## 6. Neuropilins in Liver Cancer

**Figure 2:**
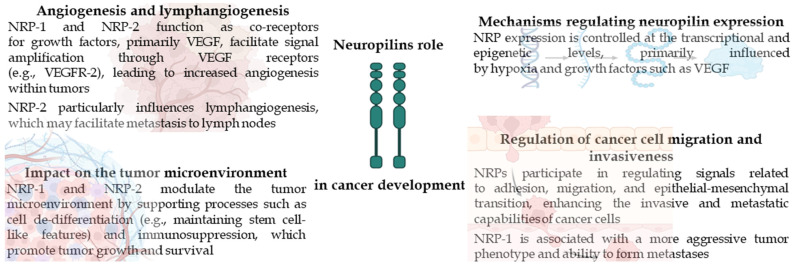
Summary of neuropilin role in cancer.

Liver cancer is also one of the top causes of cancer mortality globally. It is a very common and deadly gastrointestinal cancer, representing the 3rd most common cause of cancer incidence and the 2nd most common cause of death [40,41,42,43]. Hepatocellular carcinoma (HCC) is the most common kind of liver cancer, accounting for 90 percent of all malignant liver lesions. Chronic infections with hepatitis B and C viruses are the primary risk factors. Other causes include excessive alcohol consumption and chronic inflammation, which leads to another cause: liver cirrhosis. The long-term prognosis of patients with liver cancer remains unfavorable, with cancer recurrence being the primary cause of death [20,43,44]. The main role in the spread of HCC cells is played by the migration of single cells, which occurs through the EMT described above in the article. As a result of EMT and tumor progression, epithelial cells, such as hepatocytes, regress to an earlier period of their differentiation, i.e., the mesenchymal phenotype, characterized by an increased ability to migrate [41]. The tumor microenvironment is a significant component in the advancement of liver cancer. Hepatic stellate cells (HSCs) are among of the most significant stromal cells in liver cancers [42,45]. Studies have shown that various types of stromal cells are recruited by liver cancer, where they become activated and proliferate, thereby stimulating the metastatic potential of cancer cells [43]. However, available data on neuropilin-1 expression in HCC compared to healthy liver tissue are limited. There are only a few studies assessing the differences in expression between healthy and cancerous liver tissue. The meta-analysis performed by Fernández-Palanca P. et al. revealed that NRP-1 overexpression is mostly associated with lower survival in liver cancer patients. Moreover, increased levels of NRP-1 are connected with an increased risk of vascular invasion in liver cancer [20]. Additionally, it has been observed that increased expression of NRP-1 is associated with poor disease-free survival of patients [45]. Thus, authors have carefully suggested that NRP-1 may be an useful marker for liver cancer patient prognosis, as well as for invasive-related characteristics [20]. Recent works have also shown that NRP-1 is associated with HSC activation and the progression of liver cirrhosis. It has been found that the mechanism responsible for inhibiting the progression of liver cancer cells most likely involves blocking HSC activation, reducing the levels of transforming growth factor-β1 in the culture environment, and decreasing extracellular signal-regulated kinase activity in liver cancer cells. Therefore, NRP-1 may serve as a potential target for gene therapy in the treatment of this cancer [44]. At the molecular level, NRP-1 functions as a co-receptor for vascular endothelial growth factor (VEGF), a key mediator of tumor angiogenesis. In HCC, hypoxic conditions stabilize hypoxia-inducible factor 1-alpha (HIF-1α), which promotes VEGF expressions. The interaction between VEGF and NRP-1 enhances downstream signaling via pathways such as PI3K/AKT and MAPK/ERK, which promote proliferation, migration, and survival of HCC cells. Importantly, activation of VEGF/NRP-1 signaling can also amplify HIF-1α expression, even under normoxic conditions, thereby establishing a positive feedback loop. This loop perpetuates proangiogenic and pro-proliferative signaling in the tumor microenvironment, contributing to aggressive tumor growth and progression [45,46]. Unquestionably, neuropilins play a vital role in the regulation of angiogenesis, lymphangiogenesis, and tumor progression. However, it has been shown that in the case of HCC, the main neuropilin correlating with the mesenchymal phenotype in its cell lines is neuropilin 2. NRP-2 expression is controlled by TGFβ, which in HCC becomes a key factor driving disease progression. NRP-2 is associated with the mesenchymal phenotype in vitro, and its expression correlates with a higher stage of tumor advancement in vivo, which indicates a lower degree of cell differentiation. A meta-analysis of *EMT* gene expression studies found that TGF-β-induced EMT significantly upregulates NRP-2. On the other hand, reduced NRP-2 levels drastically impair the migratory capacity of HCC cells. Therefore, it can be concluded that NRP-2 plays an significant role in TGFβ-dependent HCC progression [41,47]. Despite the introduction of lenvatinib, resistance remains a major obstacle in HCC treatment. Tumors often bypass VEGF inhibition through alternative angiogenic pathways, increased HIF-1α, and compensatory FGFR/NRP-1 signaling. Elevated NRP-1 expression in resistant tumors supports proangiogenic activity and is associated with enhanced EMT and activation of AKT and ERK pathways, promoting tumor progression [48]. Importantly, therapeutic targeting of NRP-1 has also demonstrated significant immunomodulatory potential. It has been shown that inhibition of NRP-1 can deplete intratumoral regulatory T cells (Tregs) that mediate resistance to anti-PD-1 immunotherapy, thereby restoring T cell-mediated anti-tumor responses [49]. Furthermore, it has been found that the expression of the neuropilin NRP-2 in HCC is associated with tumor histological grade and cirrhosis, and that NRP-2-positive patients with HCC demonstrated shorter disease-free survival and overall survival compared to NRP-2-negative patients. The results of Dong X. et al. suggest that NRP-2 overexpression may be an independent factor for the prediction of unfavorable prognosis of patients with HCC. Therefore, NRP-2 could serve as a biomarker of bad prognosis, and what is more, a potential target in treating hepatocellular carcinoma [50]. The main roles of neuropilins in cancer development are presented in Figure 2.

**Figure 3:**
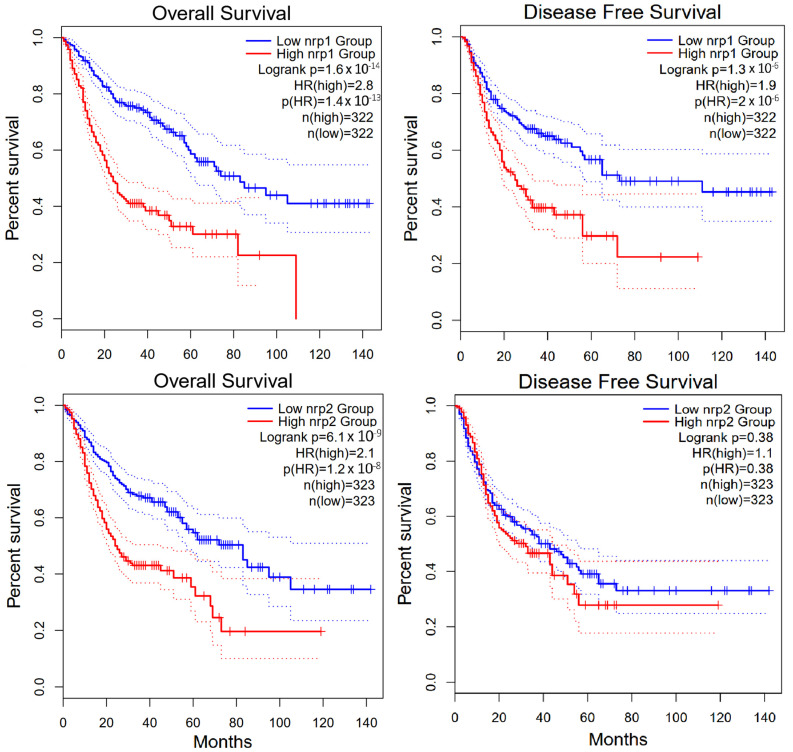
GEPIA2 software analysis of overall survival and disease free survival for NRP-1 and NRP-2 in gastrointestinal cancers [51,52].

Additional analysis concerning the review of GI cancers using GEPIA2 software [51] revealed that high NRP-1 expression is strongly associated with poorer overall survival (OS) and indicates a potential prognostic value of NRP-1 as a biomarker of poor prognosis (HR = 2.8, *p* < 0.0001). Moreover, similar to the overall survival, high NRP-1 expression is associated with a higher risk of disease relapse (HR = 1.9, *p* < 0.0001) when assessing disease-free survival (DFS). This indicates that NRP-1 may be associated not only with survival but also with tumor aggressiveness. In the case of NRP-2, high expression is also significantly associated with poorer OS (HR = 2.1, *p* < 0.0001). Although the effect is slightly lower than in the case of NRP-1, the result is still statistically significant. High NRP-2 expression was not significantly associated with DFS (HR = 1.1, *p* = 0.38) (Figure 3). This suggests that NRP-2 does not significantly affect the risk of disease relapse, although it affects overall survival [51,52].

## 7. Non-Coding RNA and NRP

In recent years, studies have shown that the expression and functions of neuropilins are strongly regulated by various types of non-coding RNAs (ncRNAs), including microRNAs (miRNAs), long non-coding RNAs (lncRNAs), and circular RNAs (circRNAs). An increasing number of reports indicate complex interactions between these molecules, which lead to modifications of NRP-1 and NRP-2-related signaling pathways in gastrointestinal cancers. For example, miR-206 has the ability to directly bind to the 3′UTR sequence of NRP-1, resulting in reduced expression of this co-receptor in gastric cancer cells and a reduction in their ability to proliferate and invade [53]. Similarly, miR-124-3p reduces NRP-1 levels in colon cancer cells, and its low level is associated with more aggressive tumor features and poorer clinical prognosis [54]. Moreover, in colorectal cancer, miR-331-3p was shown to inhibit cell invasion and migration by directly targeting the 3′ untranslated region (3′-UTR) of the NRP-2 mRNA. Overexpression of miR-331-3p suppresses epithelial–mesenchymal transition (EMT) markers and reduced NRP-2 protein levels, confirming its tumor-suppressive role in this context [55]. Furthermore, studies have shown that circNRIP1 acts as a sponge for miR-149-5p, which leads to the activation of the AKT1/mTOR pathway, promoting the proliferation, migration, and invasion of gastric cancer cells. Although the direct link between circNRIP1 and NRP-1 has not been clearly confirmed, there is evidence that other circRNAs, such as circHIPK3, can regulate NRP-1 expression through interactions with miR-653-5p and miR-338-3p, thereby affecting the migration and invasion of gastric cancer cells [56]. The presented mechanisms suggest that there is a complex, multilayered ncRNA-neuropilin regulatory network that plays an important role in the biology of gastrointestinal cancers. Understanding these interactions may provide new opportunities towards identifying prognostic and predictive biomarkers and developing molecularly targeted therapies.

## 8. Summary

Neuropilin family proteins have been shown to be connected with cancer development and may serve as therapeutic targets for gastric, liver, pancreatic, and colon cancers. Advances in neuropilin analysis have revealed multiple functional roles in cancer pathogenesis. NRP-1 is overexpressed in different malignancies, including gastric, liver, and colorectal cancer, correlating with tumor stage, metastasis, and invasion. In colorectal cancer, NRP-1 overexpression is linked to greater invasiveness and reduced survival, while NRP-2 is associated with faster tumor growth. Both neuropilins promote angiogenesis and lymphangiogenesis, aiding tumor progression. Deleting NRP-1 in preclinical models reduced migration, invasion, and angiogenesis, indicating its key role in cancer spread. Inhibitors targeting NRPs, especially in combination with anti-VEGF therapy, show potential in reducing tumor progression and metastasis. Novel studies have shown also that NRP-1 expression in gastric cancer correlates with larger tumor size, advanced stages, lymph node metastasis, making it a potential therapeutic target. Furthermore, monoclonal antibodies directed against NRP-1 have shown promising antitumor activity by inhibiting cancer cell migration and invasion. In contrast, NRP-2 has been connected with increased tumor metastasis and poor prognosis, showing that it may also be a valuable therapeutic target. Moreover, NRPs play a key role in regulation processes in liver cancer such as cell survival, tissue invasion, angiogenesis, and epithelial-mesenchymal transition (EndMT), making them therapeutic targets. Despite promising research results, their multifunctionality also poses therapeutic challenges. Neuropilins are also involved in a wide range of biological mechanisms, in pancreatic cancer, such as vascular adhesion and endocytosis regulation. The modulation of NRPs with specific inhibitors seems to be a promising approach for cancers therapy due to its role in angiogenesis, tumor progression, and fibrosis, with potential treatments including NRP-1 antagonists, CAR-T cell therapy, and siRNA delivery using polymeric nanoparticles. Overall, while NRPs offer therapeutic potential, their multifunctionality presents challenges in developing effective treatments. However, indisputably further research is required to completely grasp their function in cancer pathogenesis and optimize targeted therapies. A summary of NRP activity and effects in the course of gastrointestinal cancers is presented in Table 2.

## Figures and Tables

**Table 1:** Neuropilin 1 and Neuropilin 2 ligands.

Ligand	NRP-1	NRP-2
VEGF family proteins		
VEGF-A165	+	+
VEGF-A121	+	
VEGF-B167	+	
VEGF-C	+	+
VEGF-D	+	+
PlGF-2	+	+
VEGFR (R1/R2/R3)	+	+
Semaphorin family proteins		
SEMA3A	+	
SEMA3B, C, D, F	+	+
SEMA3G	+	
Plexin-A1 to A4, D1	+	+
Other growth factors		
TGF-β1 and LAP	+	+
TGF-β receptors (TbRI, TbRII)	+	+
FGF-1, 2, 4, 7	+	+
FGFR-1	+	
Other molecules		
Heparin	+	
HGF and c-MET	+	+
PDGF and PDGFR	+	
Integrins (α5β1, αvβ3)	+	+
Fibronectin	+	

+—the corresponding neuropilin binds to the ligand. Abbreviations: NRP-1—neuropilin 1; NRP-2—neuropilin 2; VEGF—vascular endothelial growth factor; PlGF—placental growth factor; VEGFR—vascular endothelial growth factor receptor; SEMA—semaphorin; TGF-β 1 transforming growth factor β1; LAP—latency associated protein; TbR—transforming growth factor b1 receptor; FGF—fibroblast growth factor; HGF—hepatocyte growth factor; c-MET—receptor for HGF; PDGF—platelet-derived growth factor; PDGFR—platelet-derived growth factor receptor.

**Table 2:** A summary of NRP activity and effects in the course of gastrointestinal cancers.

Neuropilin (NRP)	Cancer Type	Activity	Effect
NRP-1	Gastric cancer	Activation of VEGF and TGF-β, regulation of EMT	Increased angiogenesis, invasiveness and metastasis
	Colorectal cancer	Promotion of tumour cell migration via VEGF and PI3K/AKT signalling	Increased aggressiveness and resistance to treatment
	Pancreatic cancer	Involvement in the activation of tumour-associated fibroblasts (CAFs), regulation of the cancer microenvironment	Increased desmoplasia and cancer progression
	Liver cancer	Facilitates EMT by promoting the migration of cancer cells and enhancing their metastatic potential	Increased expression, associated with lower patient survival, higher risk of vascular invasion, and activation of HSC
NRP-2	Colorectal cancer	Regulation of VEGF-C/D signalling and lymphangiogenesis	Greater propensity for lymph node metastasis
	Pancreatic cancer	Promotes immunosuppression through effects on Treg cells	Bypassing the immune response and cancer progression
	Gastric cancer	Supports PI3K/AKT and MAPK pathways	Increased proliferation and resistance to treatment

Abbreviations: VEGF—vascular endothelial growth factor; Treg—regulatory T cells; EMT—epithelial-mesenchymal transition; PI3K—3-phosphoinositide kinase; AKT—protein kinase B; MAPK—mitogen-activated kinase; TGF-β—transforming growth factor β; HSC—hepatic stellate cells.

## Data Availability

No new data were created or analyzed in this study.
